# Effectiveness of Original Monovalent and Bivalent COVID‐19 Vaccines Against COVID‐19‐Associated Hospitalization and Severe In‐Hospital Outcomes Among Adults in the United States, September 2022–August 2023

**DOI:** 10.1111/irv.70027

**Published:** 2024-11-04

**Authors:** Jennifer DeCuir, Diya Surie, Yuwei Zhu, Adam S. Lauring, Manjusha Gaglani, Tresa McNeal, Shekhar Ghamande, Ithan D. Peltan, Samuel M. Brown, Adit A. Ginde, Aimee Steinwand, Nicholas M. Mohr, Kevin W. Gibbs, David N. Hager, Harith Ali, Anne Frosch, Michelle N. Gong, Amira Mohamed, Nicholas J. Johnson, Vasisht Srinivasan, Jay S. Steingrub, Akram Khan, Laurence W. Busse, Abhijit Duggal, Jennifer G. Wilson, Nida Qadir, Steven Y. Chang, Christopher Mallow, Jennie H. Kwon, Matthew C. Exline, Nathan I. Shapiro, Cristie Columbus, Ivana A. Vaughn, Mayur Ramesh, Basmah Safdar, Jarrod M. Mosier, Jonathan D. Casey, H. Keipp Talbot, Todd W. Rice, Natasha Halasa, James D. Chappell, Carlos G. Grijalva, Adrienne Baughman, Kelsey N. Womack, Jillian P. Rhoads, Sydney A. Swan, Cassandra Johnson, Nathaniel Lewis, Sascha Ellington, Fatimah S. Dawood, Meredith McMorrow, Wesley H. Self, for the Investigating Respiratory Viruses in the Acutely Ill (IVY) Network

**Affiliations:** ^1^ National Center for Immunization and Respiratory Diseases Centers for Disease Control and Prevention (CDC) Atlanta Georgia USA; ^2^ Department of Biostatistics Vanderbilt University Medical Center Nashville Tennessee USA; ^3^ Departments of Internal Medicine and Microbiology and Immunology University of Michigan Ann Arbor Michigan USA; ^4^ Baylor College of Medicine Baylor Scott & White Health, Temple and Dallas, Texas Temple Texas USA; ^5^ Baylor College of Medicine Baylor, Scott & White Health Temple Texas USA; ^6^ Department of Pulmonary/Critical Care Medicine, Intermountain Medical Center, Murray Utah and University of Utah Salt Lake City Utah USA; ^7^ Department of Emergency Medicine University of Colorado School of Medicine Colorado Aurora USA; ^8^ Departments of Emergency Medicine, Anesthesia Critical Care, and Epidemiology University of Iowa Carver College of Medicine Iowa City Iowa USA; ^9^ Department of Medicine Wake Forest School of Medicine Winston‐Salem North Carolina USA; ^10^ Department of Medicine Johns Hopkins University School of Medicine Baltimore Maryland USA; ^11^ Department of Emergency Medicine, Hennepin Healthcare Research Institute Hennepin Healthcare System Minneapolis Minnesota USA; ^12^ Department of Medicine, Montefiore Medical Center Albert Einstein College of Medicine Bronx New York USA; ^13^ Department of Emergency Medicine and Division of Pulmonary, Critical Care and Sleep Medicine University of Washington Seattle Washington USA; ^14^ Department of Emergency Medicine University of Washington Seattle Washington USA; ^15^ Department of Medicine Baystate Medical Center Springfield Massachusetts USA; ^16^ Department of Medicine Oregon Health and Sciences University Portland Oregon USA; ^17^ Department of Medicine Emory University Atlanta Georgia USA; ^18^ Department of Medicine Cleveland Clinic Cleveland Ohio USA; ^19^ Department of Emergency Medicine Stanford University School of Medicine Stanford California USA; ^20^ Department of Medicine University of California‐Los Angeles Los Angeles California USA; ^21^ Department of Medicine University of Miami Miami Florida USA; ^22^ Department of Medicine Washington University St. Louis Missouri USA; ^23^ Department of Medicine The Ohio State University Columbus Ohio USA; ^24^ Department of Emergency Medicine Beth Israel Deaconess Medical Center Boston Massachusetts USA; ^25^ Baylor, Scott & White Health Texas A&M University College of Medicine Dallas Texas USA; ^26^ Department of Public Health Sciences Henry Ford Health Detroit Michigan USA; ^27^ Division of Infectious Diseases Henry Ford Health Detroit Michigan USA; ^28^ Department of Emergency Medicine Yale University School of Medicine New Haven Connecticut USA; ^29^ Department of Emergency Medicine University of Arizona Tucson Arizona USA; ^30^ Department of Medicine Vanderbilt University Medical Center Nashville Tennessee USA; ^31^ Departments of Medicine and Health Policy Vanderbilt University Medical Center Nashville Tennessee USA; ^32^ Department of Pediatrics Vanderbilt University Medical Center Nashville Tennessee USA; ^33^ Department of Health Policy Vanderbilt University Medical Center Nashville Tennessee USA; ^34^ Department of Emergency Medicine Vanderbilt University Medical Center Nashville Tennessee USA; ^35^ Vanderbilt Institute for Clinical and Translational Research Vanderbilt University Medical Center Nashville Tennessee USA; ^36^ Vanderbilt Institute for Clinical and Translational Research and Department of Emergency Medicine Vanderbilt University Medical Center Nashville Tennessee USA

**Keywords:** adult, COVID‐19, COVID‐19 vaccines, hospitalization, United States

## Abstract

**Background:**

Assessments of COVID‐19 vaccine effectiveness are needed to monitor the protection provided by updated vaccines against severe COVID‐19. We evaluated the effectiveness of original monovalent and bivalent (ancestral strain and Omicron BA.4/5) COVID‐19 vaccination against COVID‐19‐associated hospitalization and severe in‐hospital outcomes.

**Methods:**

During September 8, 2022 to August 31, 2023, adults aged ≥ 18 years hospitalized with COVID‐19‐like illness were enrolled at 26 hospitals in 20 US states. Using a test‐negative case–control design, we estimated vaccine effectiveness (VE) with multivariable logistic regression adjusted for age, sex, race/ethnicity, admission date, and geographic region.

**Results:**

Among 7028 patients, 2924 (41.6%) were COVID‐19 case patients, and 4104 (58.4%) were control patients. Compared to unvaccinated patients, absolute VE against COVID‐19‐associated hospitalization was 6% (−7%–17%) for original monovalent doses only (median time since last dose [IQR] = 421 days [304–571]), 52% (39%–61%) for a bivalent dose received 7–89 days earlier, and 13% (−10%–31%) for a bivalent dose received 90–179 days earlier. Absolute VE against COVID‐19‐associated invasive mechanical ventilation or death was 51% (34%–63%) for original monovalent doses only, 61% (35%–77%) for a bivalent dose received 7–89 days earlier, and 50% (11%–71%) for a bivalent dose received 90–179 days earlier.

**Conclusion:**

Bivalent vaccination provided protection against COVID‐19‐associated hospitalization and severe in‐hospital outcomes within 3 months of receipt, followed by a decline in protection to a level similar to that remaining from previous original monovalent vaccination by 3–6 months. These results underscore the benefit of remaining up to date with recommended COVID‐19 vaccines.

## 1 Introduction

In December 2020, monovalent COVID‐19 vaccines designed against the ancestral strain of SARS‐CoV‐2 (original monovalent vaccines) were introduced in the United States to prevent COVID‐19‐associated morbidity and mortality. Although these vaccines successfully prevented COVID‐19‐associated hospitalization and death [1, 2], their effectiveness declined over time due to several factors, including waning vaccine‐induced immunity and the emergence of the immune evasive Omicron variant [3, 4]. To address these factors, on September 1, 2022, the Advisory Committee on Immunization Practices (ACIP) at the US Centers for Disease Control and Prevention (CDC) recommended a bivalent mRNA COVID‐19 dose, designed to protect against both the ancestral strain and Omicron BA.4/5 lineages, for persons who had completed at least an original monovalent COVID‐19 primary series [5]. With implementation of this recommendation, the original monovalent mRNA COVID‐19 vaccines were no longer recommended for use as booster doses in the United States [6]. The following year, in September 2023, the ACIP recommended vaccination with a 2023–2024 COVID‐19 vaccine, designed against the Omicron XBB.1.5 lineage, and it is anticipated that periodic updates to COVID‐19 vaccine composition will continue to occur [7].

Assessments of COVID‐19 vaccines against a variety of clinical outcomes are needed to monitor the effectiveness of COVID‐19 vaccination programs and to communicate the value of receiving COVID‐19 vaccines. We evaluated the effectiveness of the original monovalent and bivalent (ancestral strain and Omicron BA.4/5) vaccination against COVID‐19‐associated hospitalization and severe in‐hospital outcomes. The results on bivalent mRNA COVID‐19 vaccine effectiveness were stratified by time since bivalent dose receipt to examine durability of protection.

## 2 Methods

### 2.1 Setting and Design

This test‐negative case–control analysis was conducted by the Investigating Respiratory Viruses in the Acutely Ill (IVY) Network, which consisted of 26 hospitals in 20 US states in collaboration with the CDC. The current analysis included adults admitted to IVY Network hospitals from September 8, 2022, to August 31, 2023. Vaccine effectiveness for the prevention of COVID‐19‐associated hospitalization and severe in‐hospital outcomes was estimated for the original monovalent and bivalent COVID‐19 vaccines authorized for use in the United States during the analysis period. These activities were determined to be public health surveillance with waiver of informed consent by institutional review boards at CDC and each enrolling site and were conducted in accordance with applicable federal law and CDC policy (45 C.F.R. part 46.102(l)(2), 21 C.F.R. part 56; 42 U.S.C. §241(d); 5 U.S.C. §552a; 44 U.S.C. §3501 et seq).

### 2.2 Participants

Site personnel prospectively enrolled patients aged ≥ 18 years admitted to IVY Network hospitals who met a COVID‐19‐like illness case definition and received SARS‐CoV‐2 clinical testing. COVID‐19‐like illness was defined as ≥ 1 of the following five signs and symptoms: fever, cough, shortness of breath, new or worsening findings on chest imaging consistent with pneumonia, or hypoxemia (defined as an oxygen saturation [SpO_2_] < 92% or supplemental oxygen use for patients without chronic oxygen needs or escalation of oxygen therapy for patients on chronic supplemental oxygen). Case patients tested positive for SARS‐CoV‐2 by nucleic acid amplification test or antigen test within 10 days of illness onset and within 3 days of hospital admission. Control patients tested negative for SARS‐CoV‐2 by real‐time reverse transcription polymerase chain reaction (RT‐PCR) within 10 days of illness onset and within 3 days of hospital admission. Case or control status was determined by results of both clinical SARS‐CoV‐2 testing at the admitting hospital and standardized central laboratory testing for SARS‐CoV‐2 by RT‐PCR. Patients who tested positive for SARS‐CoV‐2 by either clinical testing or central laboratory testing were classified as cases, whereas patients who tested negative by both clinical and central laboratory testing were classified as controls. Case patients who tested positive for influenza or RSV were excluded from analyses, whereas control patients who tested positive for influenza were also excluded due to potential correlation between COVID‐19 and influenza vaccination behaviors [8]. Case and control patients were enrolled by admission date within 2 weeks of one another at each site in approximately a 1:1 ratio and were not matched on patient‐level characteristics.

### 2.3 Data Collection

Trained personnel at enrolling sites collected data on patient demographics through patient or proxy interview and on chronic medical conditions and severe in‐hospital outcomes through medical chart abstraction. Chronic medical conditions were grouped into nine categories: cardiovascular, pulmonary, renal, endocrine, gastrointestinal, hematologic, neurologic, autoimmune, and immunocompromising (see Appendix S2). COVID‐19 vaccination status was ascertained using hospital electronic medical records, state vaccination registries, vaccination cards (when available), and patient or proxy interview. Patients were followed from admission until discharge, death, or hospital day 28 (whichever occurred first).

### 2.4 Classification of Vaccination Status

To assess original monovalent and bivalent vaccine effectiveness, patients were classified into four vaccination status groups: (1) unvaccinated (no COVID‐19 vaccine doses received), (2) vaccinated with original monovalent doses only (receipt of any combination of one to four doses of original monovalent vaccine: mRNA‐1273 [Moderna], BNT162b2 [Pfizer‐BioNTech], Ad26.COV2.S [Janssen], NVX‐CoV2373 [Novavax]), (3) vaccinated with one bivalent mRNA dose 7–89 days before illness onset, and (4) vaccinated with one bivalent mRNA dose 90–179 days before illness onset. Patients were classified as bivalent vaccinated if they received one dose of either mRNA‐1273.222 (Moderna) or BNT1262b2 bivalent (Pfizer‐BioNTech), regardless of the number of previous original monovalent doses received. Patients were excluded if they received > 1 bivalent dose; if they received any COVID‐19 vaccine dose < 7 days before illness onset; if they received only one dose of original monovalent Moderna, Pfizer, or Novavax vaccine; or if they had an immunocompromising condition (defined in Appendix S2). Estimates of bivalent vaccine effectiveness ≥ 180 days after dose receipt lacked sufficient precision for reporting; therefore, patients who received a bivalent dose ≥ 180 days before illness onset were also excluded.

### 2.5 Clinical Outcomes

COVID‐19‐associated hospitalization was defined as hospital admission with COVID‐19‐like illness and laboratory‐confirmed SARS‐CoV‐2 infection as described above. Additional outcomes were also included to assess original monovalent and bivalent vaccine effectiveness against severe COVID‐19‐associated disease through hospital day 28. These outcomes were (1) supplemental oxygen therapy, defined as any receipt of supplemental oxygen for patients with no chronic oxygen use or escalation of oxygen use for patients on chronic supplemental oxygen; (2) advanced respiratory support, defined as new receipt of high‐flow nasal cannula (HFNC), noninvasive ventilation (NIV), or invasive mechanical ventilation (IMV); (3) acute organ failure, defined as either respiratory failure (new receipt of HFNC, NIV, or IMV), cardiovascular failure (receipt of vasopressors), or renal failure (new receipt of renal replacement therapy); (4) intensive care unit (ICU) admission; and (5) IMV or death. Patients on home IMV were not eligible for the supplemental oxygen therapy or advanced respiratory support outcomes (outcomes are fully described in Appendix S2).

### 2.6 Molecular Diagnosis and Sequencing

Nasal swabs were obtained from enrolled patients and tested for SARS‐CoV‐2, influenza, and RSV by RT‐PCR at a central laboratory at Vanderbilt University Medical Center (Nashville, Tennessee). All specimens that tested positive for SARS‐CoV‐2 by central RT‐PCR testing were submitted to the University of Michigan (Ann Arbor, Michigan) for viral whole genome sequencing. Detailed laboratory methods are described in Appendix S2. SARS‐CoV‐2 lineages were considered predominant if they were detected in ≥ 50% of sequenced specimens during a given admission week.

### 2.7 Statistical Analysis

Original monovalent and bivalent vaccine effectiveness against COVID‐19‐associated hospitalization was calculated using multivariable logistic regression, in which the odds of COVID‐19 vaccination were compared between COVID‐19 case patients and control patients. Estimates of vaccine effectiveness were generated using two different comparator groups. First, absolute vaccine effectiveness was calculated for both bivalent and original monovalent vaccines using unvaccinated patients as the reference group. Second, relative vaccine effectiveness was calculated for bivalent vaccines using patients who received original monovalent vaccination only as the reference group. Logistic regression models were adjusted for age (18–49, 50–64, and ≥ 65), sex (male and female), self‐reported race and Hispanic ethnicity (non‐Hispanic white, non‐Hispanic Black, Hispanic or Latino, non‐Hispanic other race, and other), admission date in biweekly intervals, and US Department of Health and Human Services region of the admitting hospital. Post hoc, we evaluated other variables as potential covariates, including the number of chronic medical condition categories and known previous Omicron infection, defined as either self‐reported or documented SARS‐CoV‐2 infection on or after December 21, 2021. None of these other variables resulted in an absolute change in the adjusted odds ratio of > 5% when added to the prespecified model and were not included in the final models (Table S1) [9]. Vaccine effectiveness was calculated as (1 − adjusted odds ratio) × 100%.

Overall estimates of original monovalent and bivalent vaccine effectiveness against COVID‐19‐associated hospitalization were stratified by age (18–64 and ≥ 65 years), number of chronic medical condition categories (< 3, ≥ 3), and bivalent vaccine product type (Moderna, Pfizer). Analyses assessing original monovalent and bivalent vaccine effectiveness against COVID‐19‐associated in‐hospital outcomes were conducted using the methods described for COVID‐19‐associated hospitalization, with cases limited to those who met each severe in‐hospital outcome definition. Analyses were conducted using SAS (Version 9.4; SAS Institute).

## 3 Results

### 3.1 Description of Participants in Vaccine Effectiveness Analyses

Between September 8, 2022, and August 31, 2023, 8783 immunocompetent patients admitted with COVID‐19‐like illness were enrolled in the IVY Network from 26 hospitals in 20 US states (Figure S1). A total of 1755 (20.0%) patients met exclusion criteria and were removed from the analysis. Among the remaining 7028 patients, 2924 (41.6%) were COVID‐19 case patients, and 4104 (58.4%) were control patients (Table 1). The median age was 67 years (interquartile range [IQR] 56–78), 3573 (50.8%) patients were female, 1606 (22.9%) were non‐Hispanic Black, and 802 (11.4%) were Hispanic. A total of 1545 (22.0%) patients were unvaccinated, 4191 (59.6%) were vaccinated with original monovalent doses only, 647 (9.2%) were vaccinated with one bivalent dose 7–89 days before illness onset, and 645 (9.2%) were vaccinated with one bivalent dose 90–179 days before illness onset. Compared with patients who received either original monovalent or bivalent vaccine doses, unvaccinated patients were younger (*p* < 0.0001) and more likely to be non‐Hispanic Black (*p* = 0.0338) or Hispanic (*p* = 0.0002).

**TABLE 1:** Characteristics of adults by vaccination and COVID‐19 status admitted to one of 26 hospitals in 20 US states during September 8, 2022–August 31, 2023. Values are numbers (column percentages) unless stated otherwise.

Characteristics	Total (*n* = 7028)	Vaccination status				COVID‐19 status	
		Unvaccinated (*n* = 1545)	Original monovalent only (*n* = 4191)	Bivalent 7–89 days earlier (*n* = 647)	Bivalent 90–179 days earlier (*n* = 645)	COVID‐19 case patients (*n* = 2924)	Control patients (*n* = 4104)
Median (IQR) age (years)	67 (56–78)	59 (43–72)	68 (57–78)	72 (62–80)	72 (64–81)	70 (58–80)	66 (54–76)
Age group (years)							
18–49	1257 (17.9)	524 (33.9)	649 (15.5)	49 (7.6)	35 (5.4)	482 (16.5)	775 (18.9)
50–64	1759 (25.0)	408 (26.4)	1086 (25.9)	133 (20.6)	132 (20.5)	620 (21.2)	1139 (27.8)
≥ 65	4012 (57.1)	613 (39.7)	2456 (58.6)	465 (71.9)	478 (74.1)	1822 (62.3)	2190 (53.4)
Female	3573 (50.8)	770 (49.8)	2169 (51.8)	314 (48.5)	320 (49.6)	1500 (51.3)	2073 (50.5)
Race and ethnicity							
White, non‐Hispanic	4092 (58.2)	814 (52.7)	2424 (57.8)	415 (64.1)	439 (68.1)	1712 (58.6)	2380 (58.0)
Black or African American, non‐Hispanic	1606 (22.9)	384 (24.9)	968 (23.1)	129 (19.9)	125 (19.4)	635 (21.7)	971 (23.7)
Hispanic or Latino, any race	802 (11.4)	218 (14.1)	492 (11.7)	52 (8.0)	40 (6.2)	356 (12.2)	446 (10.9)
Other race, non‐Hispanic^a^	237 (3.4)	57 (3.7)	143 (3.4)	18 (2.8)	19 (3.0)	100 (3.4)	137 (3.3)
Other^b^	291 (4.1)	72 (4.7)	164 (3.9)	33 (5.1)	22 (3.4)	121 (4.1)	170 (4.1)
HHS region^c^							
1	1393 (19.8)	225 (14.6)	840 (20.0)	152 (23.5)	176 (27.3)	628 (21.5)	765 (18.6)
2	408 (5.8)	112 (7.3)	252 (6.0)	27 (4.2)	17 (2.6)	156 (5.3)	252 (6.1)
3	236 (3.4)	23 (1.5)	161 (3.8)	22 (3.4)	30 (4.7)	94 (3.2)	142 (3.5)
4	1053 (15.0)	265 (17.2)	650 (15.5)	69 (10.7)	69 (10.7)	444 (15.2)	609 (14.8)
5	1110 (15.8)	242 (15.7)	643 (15.3)	122 (18.9)	103 (16.0)	456 (15.6)	654 (15.9)
6	753 (10.7)	202 (13.1)	475 (11.3)	43 (6.7)	33 (5.1)	339 (11.6)	414 (10.1)
7	420 (6.0)	109 (7.1)	243 (5.8)	35 (5.4)	33 (5.1)	166 (5.7)	254 (6.2)
8	1038 (14.8)	235 (15.2)	574 (13.7)	108 (16.7)	121 (18.8)	382 (13.1)	656 (16.0)
9	389 (5.5)	78 (5.1)	235 (5.6)	35 (5.4)	41 (6.4)	158 (5.4)	231 (5.6)
10	228 (3.2)	54 (3.5)	118 (2.8)	34 (5.3)	22 (3.4)	101 (3.5)	127 (3.1)
No. of chronic medical condition categories, median (IQR)^d^	2 (1–3)	2 (1–3)	2 (1–3)	2 (1–3)	2 (1–3)	2 (1–3)	2 (1–3)
Previous SARS‐CoV‐2 infection^e^							
Any previous SARS‐CoV‐2 infection	1445 (20.6)	307 (19.9)	899 (21.5)	117 (18.1)	122 (18.9)	491 (16.8)	954 (23.3)
Previous Omicron variant infection	929 (13.2)	191 (12.4)	579 (13.8)	74 (11.4)	85 (13.2)	284 (9.7)	645 (15.7)
COVID‐19 status							
Case patients	2924 (41.6)	660 (42.7)	1811 (43.2)	184 (28.4)	269 (41.7)	2924 (100.0)	—
Control patients	4104 (58.4)	885 (57.3)	2380 (56.8)	463 (71.6)	376 (58.3)	—	4104 (100.0)
Vaccination status							
Unvaccinated	1545 (22.0)	1545 (100.0)	—	—	—	660 (22.6)	885 (21.6)
Monovalent only	4191 (59.6)	—	4191 (100.0)	—	—	1811 (61.9)	2380 (58.0)
Bivalent 7–89 days earlier	647 (9.2)	—	—	647 (100.0)	—	184 (6.3)	463 (11.3)
Bivalent 90–179 days earlier	645 (9.2)	—	—	—	645 (100.0)	269 (9.2)	376 (9.2)
Bivalent vaccine product received^f^							
Moderna	396 (30.7)	—	—	190 (29.4)	206 (31.9)	132 (29.1)	264 (31.5)
Pfizer	896 (69.4)	—	—	457 (70.6)	439 (68.1)	321 (70.9)	575 (68.5)

Abbreviations: HHS = U.S. Department of Health and Human Services, IQR = interquartile range.

^a^ “Other race, non‐Hispanic” includes Asian, Native American or Alaska Native, and Native Hawaiian or other Pacific Islander; these groups were combined due to small counts.

^b^ “Other” includes patients who self‐reported their race and ethnicity as “Other” and those for whom race and ethnicity were unknown.

^c^ Hospitals by HHS region included *Region 1*: Baystate Medical Center (Springfield, Massachusetts), Beth Israel Deaconess Medical Center (Boston, Massachusetts), and Yale University (New Haven, Connecticut); *Region 2*: Montefiore Medical Center (New York, New York); *Region 3*: Johns Hopkins Hospital (Baltimore, Maryland); *Region 4*: Emory University Medical Center (Atlanta, Georgia), University of Miami Medical Center (Miami, Florida), Vanderbilt University Medical Center (Nashville, Tennessee), and Wake Forest University Baptist Medical Center (Winston‐Salem, North Carolina); *Region 5*: Cleveland Clinic (Cleveland, Ohio), Hennepin County Medical Center (Minneapolis, Minnesota), Henry Ford Health (Detroit, Michigan), The Ohio State University Wexner Medical Center (Columbus, Ohio), and University of Michigan Hospital (Ann Arbor, Michigan); *Region 6*: Baylor Scott & White Medical Center (Temple, Texas) and Baylor University Medical Center (Dallas, Texas); *Region 7*: Barnes‐Jewish Hospital (St. Louis, Missouri) and University of Iowa Hospitals (Iowa City, Iowa); *Region 8*: Intermountain Medical Center (Murray, Utah), UCHealth University of Colorado Hospital (Aurora, Colorado), and University of Utah (Salt Lake City, Utah); *Region 9*: Stanford University Medical Center (Stanford, California), Ronald Reagan UCLA Medical Center (Los Angeles, California), and University of Arizona Medical Center (Tucson, Arizona); and *Region 10*: Oregon Health & Science University Hospital (Portland, Oregon) and University of Washington (Seattle, Washington).

^d^ Chronic medical condition categories include autoimmune, cardiovascular, endocrine, gastrointestinal, hematologic, neurologic, pulmonary, and renal diseases.

^e^ Previous SARS‐CoV‐2 infection was defined as any self‐reported or documented SARS‐CoV‐2 infection that occurred before the episode of illness for which the patient was enrolled into the IVY Network. Previous Omicron infection was defined as any self‐reported or documented SARS‐CoV‐2 infection that occurred from December 26, 2021, until the episode of illness for which the patient was enrolled into the IVY Network.

^f^ Proportion denominators restricted to bivalent recipients only.

Viral whole genome sequencing was completed for 1779 of the 2924 case patients (60.8%), and a SARS‐CoV‐2 lineage was successfully identified for 1646 (56.3%), all of whom were infected with an Omicron lineage. BA.4/5 was the predominant lineage from September 8, 2022, until November 26, 2022; BQ.1 was predominant from December 4, 2022, to December 31, 2022; and XBB.1.5 was predominant from January 22, 2023, to May 27, 2023 (Figure S2). No single SARS‐CoV‐2 lineage was predominant from May 28, 2023, to August 31, 2023.

### 3.2 Vaccine Effectiveness Against COVID‐19‐Associated Hospitalization

**FIGURE 1:**
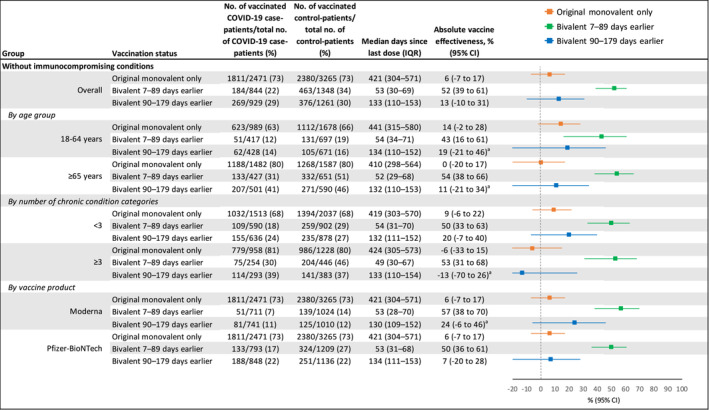
Absolute COVID‐19 vaccine effectiveness against COVID‐19‐associated hospitalization among adults in the United States during September 8, 2022–August 31, 2023, including effectiveness of original monovalent vaccines only, bivalent mRNA vaccines received 7–89 days before illness onset, and bivalent mRNA vaccines received 90–179 days before illness onset compared to unvaccinated patients. CI = confidence interval, IQR = interquartile range. ^a^Some estimates are imprecise, which might be due to a relatively small number of persons in each level of vaccination or case status. This imprecision indicates that the actual vaccine effectiveness could be substantially different from the point estimate shown, and estimates should therefore be interpreted with caution.

The absolute effectiveness of original monovalent vaccination only against COVID‐19‐associated hospitalization was 6% (95% CI −7%–17%), with a median time since last dose of 421 days (IQR 304–571). Bivalent vaccination received 7–89 days before illness onset provided significantly higher protection against hospitalization than original monovalent vaccination only, with an absolute vaccine effectiveness of 52% (39%–61%) and a relative vaccine effectiveness of 48% (36%–57%). After 90–179 days, absolute effectiveness waned to 13% (−10%–31%), and relative effectiveness waned to 17% (−1%–31%) (Figures 1 and S3).

Bivalent vaccination provided increased protection against COVID‐19‐associated hospitalization 7–89 days after dose receipt compared to original monovalent vaccination only, followed by a decline in point estimates of vaccine effectiveness by 90–179 days across most subgroups, including patients aged ≥ 65 years, patients with comorbidities in < 3 and ≥ 3 chronic condition categories, Moderna bivalent recipients, and Pfizer bivalent recipients (Figures 1 and S3).

### 3.3 Vaccine Effectiveness Against COVID‐19‐Associated Severe In‐Hospital Outcomes

Among 2924 case patients, 632 (21.6%) experienced acute organ failure, 514 (17.8%) were admitted to an ICU, and 281 (9.6%) received IMV or died (Table 2). Among 2918 case patients not on home IMV, 1775 (60.8%) received supplemental oxygen therapy, and 568 (19.5%) received advanced respiratory support. Compared with case patients who received either original monovalent or bivalent vaccination, unvaccinated case patients were more likely to have advanced respiratory support (*p* = 0.0005), acute organ failure (*p* = 0.0065), ICU admission (*p* < 0.0001), and IMV or death (*p* < 0.0001).

**TABLE 2:** Severe in‐hospital outcomes among adults admitted to hospital with COVID‐19 during September 8, 2022–August 31, 2023, by vaccination status. Values are numbers (percentages) of total.

Clinical outcome	No. with outcome/total	No. unvaccinated with outcome/total unvaccinated	No. original monovalent only with outcome/total original monovalent only	No. bivalent 7–89 days earlier with outcome/total bivalent 7–89 days earlier	No. bivalent 90–179 days earlier with outcome/total bivalent 90–179 days earlier
Supplemental oxygen therapy^a^	1775/2918 (60.8)	406/658 (61.7)	1086/1807 (60.1)	120/184 (65.2)	163/269 (60.6)
Advanced respiratory support^b^	568/2918 (19.5)	159/658 (24.2)	323/1807 (17.9)	34/184 (18.5)	52/269 (19.3)
Acute organ failure^c^	632/2924 (21.6)	168/660 (25.5)	371/1811 (20.8)	39/184 (21.2)	54/269 (20.1)
ICU admission	514/2924 (17.8)	153/660 (23.2)	281/1811 (15.5)	38/184 (20.7)	42/269 (15.6)
IMV or death	281/2924 (9.6)	92/660 (13.9)	141/1811 (7.8)	25/184 (13.4)	23/269 (8.6)

Abbreviations: ICU = intensive care unit, IMV = invasive mechanical ventilation.

^a^ Supplemental oxygen therapy was defined as any receipt of supplemental oxygen for patients with no chronic oxygen needs or escalation of respiratory support for patients on chronic oxygen at baseline. Patients on home IMV were not eligible for this outcome.

^b^ Advanced respiratory support was defined as new receipt of high‐flow nasal cannula, non‐invasive ventilation, or IMV. Patients on home IMV were not eligible for this outcome.

^c^ Acute organ failure was defined as respiratory failure (new receipt of high‐flow nasal cannula, noninvasive ventilation, or IMV), cardiovascular failure (receipt of vasopressors), or renal failure (new receipt of renal replacement therapy).

**FIGURE 2:**
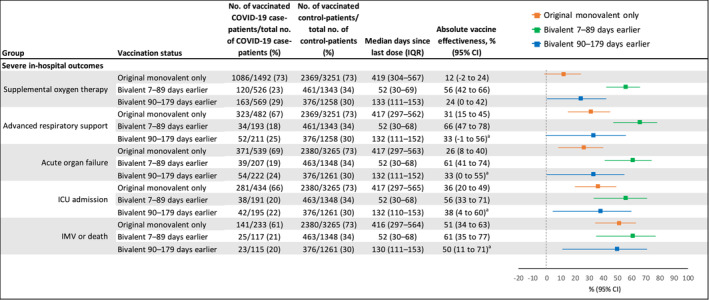
Absolute COVID‐19 vaccine effectiveness against COVID‐19‐associated severe in‐hospital outcomes among adults in the United States during September 8, 2022–August 31, 2023, including effectiveness of original monovalent vaccines only, mRNA bivalent vaccines received 7–89 days before illness onset, and mRNA bivalent vaccines received 90–179 days before illness onset compared to unvaccinated patients. CI = confidence interval, ICU = intensive care unit, IMV = invasive mechanical ventilation, IQR = interquartile range. ^a^Some estimates are imprecise, which might be due to a relatively small number of persons in each level of vaccination or case status. This imprecision indicates that the actual VE could be substantially different from the point estimate shown, and estimates should therefore be interpreted with caution.

The absolute effectiveness of original monovalent vaccination only against COVID‐19‐associated supplemental oxygen therapy (12%, −2%–24%) was similar to that against COVID‐19‐associated hospitalization (6%, −7%–17%) (Figures 1 and 2). Point estimates of the absolute effectiveness of original monovalent vaccination only generally increased with outcome severity, up to 51% (34%–63%) against IMV or death (median time since last dose = 416 days, IQR = 297–564). Compared to the absolute effectiveness of original monovalent doses only, the absolute effectiveness of a bivalent dose received 7–89 days before illness onset was higher against COVID‐19‐associated supplemental oxygen therapy (original monovalent: 12%, −2%–24% vs. bivalent: 56%, 42%–66%), advanced respiratory support (original monovalent: 31%, 15%–45% vs. bivalent: 66%, 47%–78%), and acute organ failure (original monovalent: 26%, 8%–40% vs. bivalent: 61%, 41%–74%) (Figure 2). Estimates of absolute effectiveness for original monovalent vaccination and bivalent vaccination received 7–89 days before illness onset were similar against ICU admission (original monovalent: 36%, 20%–49% vs. bivalent: 56%, 33%–71%) and IMV or death (original monovalent: 51%, 34%–63% vs. bivalent: 61%, 35%–77%). By 90–179 days after vaccination, point estimates of bivalent vaccine effectiveness against each severe outcome had declined to a level similar to that for original monovalent vaccination only. Trends were similar when assessing relative bivalent effectiveness (Figure S4).

## 4 Discussion

In this analysis of adults admitted to 26 US hospitals between September 8, 2022, and August 31, 2023, no protection against COVID‐19‐associated hospitalization remained from original monovalent vaccination only, for which the median time since last dose was > 1 year. In contrast, bivalent mRNA COVID‐19 vaccination provided protection against COVID‐19‐associated hospitalization within 3 months of dose receipt, when compared to both unvaccinated patients and patients who received original monovalent doses only. However, protection from bivalent vaccination waned by 3–6 months after dose receipt. These findings were consistent across key subgroups, including patients aged ≥ 65 years and patients with multiple comorbidities. Of note, original monovalent vaccination continued to provide durable protection against the most severe in‐hospital outcomes, including IMV or death. Bivalent vaccination increased protection against certain severe in‐hospital outcomes within 3 months of dose receipt before declining to a level of protection similar to that remaining from previous original monovalent vaccination. These results support staying up to date with recommended COVID‐19 vaccines to optimize protection against both COVID‐19‐associated hospitalization and severe in‐hospital outcomes.

Our findings are consistent with studies from the United States, the United Kingdom, and Finland showing that bivalent COVID‐19 vaccination provided protection against COVID‐19‐associated hospitalization, followed by waning within 6 months of dose receipt [10, 11, 12, 13]. A CDC report on US adults aged ≥ 18 years found that the absolute effectiveness of a bivalent vaccine dose against COVID‐19‐associated hospitalization was 62% (57%–67%) at 7–59 days after vaccination and declined to 24% (12%–33%) after 120–179 days, similar to our results [10]. Declining estimates of bivalent vaccine effectiveness against COVID‐19‐associated hospitalization may be explained by a number of factors, including the emergence of immune evasive lineages and waning vaccine‐induced immunity.

Our results also provide important information regarding the effectiveness and durability of COVID‐19 vaccination against the most severe in‐hospital outcomes. When compared to unvaccinated patients, bivalent vaccination provided protection against all in‐hospital outcomes assessed in this analysis, including acute organ failure, ICU admission, and IMV or death, and provided additional protection beyond that remaining from previous original monovalent vaccination for some outcomes. However, protection from bivalent vaccination declined by 3–6 months to a level similar to that remaining from previous original monovalent vaccination. Although original monovalent vaccination provided residual protection against severe in‐hospital outcomes > 1 year after receipt of the last dose, possibly mediated by long‐lasting memory B‐ and T‐cell responses [14, 15, 16], the longer term durability of protection from original monovalent vaccination amidst continued viral evolution is unclear. Taken collectively, our findings demonstrated an added benefit of updated COVID‐19 vaccination to optimize protection against severe in‐hospital outcomes irrespective of prior COVID‐19 vaccination status, consistent with other studies of bivalent vaccine effectiveness [11, 17, 18, 19].

As with all observational research, this analysis had limitations. Because original monovalent and bivalent COVID‐19 vaccines were not simultaneously available as booster doses in the United States, a direct comparison of original monovalent and bivalent vaccine effectiveness against COVID‐19‐associated hospitalization and severe outcomes was not possible during the study period. Although case patients tested positive for SARS‐CoV‐2 and met criteria for COVID‐19‐like illness, some may have been hospitalized for reasons other than COVID‐19, which could have biased vaccine effectiveness estimates toward the null. The measures of prior SARS‐CoV‐2 infection available for inclusion in this analysis likely underestimated the proportion of patients with prior infection in our sample and were not included in vaccine effectiveness models, as described above. Previous studies have demonstrated that prior infection is highly protective against severe outcomes and may be more prevalent among patients who are unvaccinated or have a remote history of vaccination compared to recent vaccinees [20, 21], resulting in lower measured vaccine effectiveness. Therefore, estimates of COVID‐19 vaccine effectiveness should be interpreted as the incremental benefit of vaccination beyond protection provided by population immunity. Although vaccine effectiveness estimates were adjusted for patient‐level demographic characteristics, calendar time, and geographic region, residual confounding from other factors, including the receipt of COVID‐19 antiviral treatments, is also possible. Finally, sample size limitations resulted in wide confidence intervals for some effectiveness estimates and prevented calculation of original monovalent and bivalent vaccine effectiveness against death alone.

In light of waning COVID‐19 vaccine effectiveness documented in this and other studies, it is anticipated that COVID‐19 vaccines will be periodically updated to optimize antigenic match with lineages predicted to circulate. Receipt of a recommended updated COVID‐19 vaccine may boost waned vaccine‐induced immunity against severe COVID‐19 outcomes and improve protection against emerging lineages. In June 2024, the World Health Organization Technical Advisory Group on COVID‐19 Vaccine Composition recommended an update to COVID‐19 vaccines for the fall of 2024 to a monovalent JN.1 lineage antigen [22]. Future monitoring of SARS‐CoV‐2 epidemiology, the burden of disease in high‐risk groups, and the effectiveness of updated COVID‐19 vaccines will be critical to informing policy on the need for regular revaccination against COVID‐19. Findings from this analysis highlight the importance of staying up to date with recommended COVID‐19 vaccines to optimize protection against both hospitalization and severe in‐hospital outcomes due to COVID‐19.

## Author Contributions

**Jennifer DeCuir:** conceptualization, methodology, formal analysis, writing–original draft, writing–review and editing, visualization. **Diya Surie:** conceptualization, methodology, writing–original draft, writing–review and editing, project administration, supervision, funding acquisition. **Yuwei Zhu:** writing–review and editing, supervision, formal analysis. **Adam S. Lauring:** writing–review and editing, data curation, funding acquisition. **Manjusha Gaglani:** data curation, funding acquisition, writing–review and editing. **Tresa McNeal:** writing–review and editing, data curation. **Shekhar Ghamande:** writing–review and editing, data curation. **Ithan D. Peltan:** data curation, writing–review and editing, funding acquisition. **Samuel M. Brown:** data curation, writing–review and editing, funding acquisition. **Adit A. Ginde:** data curation, writing–review and editing, funding acquisition. **Aimee Steinwand:** data curation, writing–review and editing. **Nicholas M. Mohr:** funding acquisition, writing–review and editing, data curation. **Kevin W. Gibbs:** funding acquisition, writing–review and editing, data curation. **David N. Hager:** funding acquisition, writing–review and editing, data curation. **Harith Ali:** writing–review and editing, data curation. **Anne Frosch:** funding acquisition, writing–review and editing, data curation. **Michelle N. Gong:** funding acquisition, writing–review and editing, data curation. **Amira Mohamed:** writing–review and editing, data curation. **Nicholas J. Johnson:** writing–review and editing, data curation, funding acquisition. **Vasisht Srinivasan:** writing–review and editing, data curation. **Jay S. Steingrub:** writing–review and editing, data curation, funding acquisition. **Akram Khan:** writing–review and editing, data curation. **Laurence W. Busse:** writing–review and editing, data curation, funding acquisition. **Abhijit Duggal:** funding acquisition, writing–review and editing, data curation. **Jennifer G. Wilson:** funding acquisition, writing–review and editing, data curation. **Nida Qadir:** writing–review and editing, data curation, funding acquisition. **Steven Y. Chang:** writing–review and editing, data curation, funding acquisition. **Christopher Mallow:** funding acquisition, writing–review and editing, data curation. **Jennie H. Kwon:** funding acquisition, writing–review and editing, data curation. **Matthew C. Exline:** funding acquisition, writing–review and editing, data curation. **Nathan I. Shapiro:** funding acquisition, writing–review and editing, data curation. **Cristie Columbus:** writing–review and editing, data curation. **Ivana A. Vaughn:** writing–review and editing, data curation, funding acquisition. **Mayur Ramesh:** writing–review and editing, data curation. **Basmah Safdar:** funding acquisition, writing–review and editing, data curation. **Jarrod M. Mosier:** funding acquisition, writing–review and editing, data curation. **Jonathan D. Casey:** writing–review and editing, data curation. **H. Keipp Talbot:** writing–review and editing, data curation. **Todd W. Rice:** writing–review and editing, data curation. **Natasha Halasa:** writing–review and editing, data curation. **James D. Chappell:** writing–review and editing, data curation. **Carlos G. Grijalva:** writing–review and editing, data curation. **Adrienne Baughman:** writing–review and editing, data curation. **Kelsey N. Womack:** writing–review and editing, data curation. **Jillian P. Rhoads:** writing–review and editing, data curation. **Sydney A. Swan:** writing–review and editing, data curation. **Cassandra Johnson:** writing–review and editing, data curation. **Nathaniel Lewis:** writing–review and editing, methodology. **Sascha Ellington:** writing–review and editing, methodology. **Fatimah S. Dawood:** writing–review and editing, supervision, project administration, methodology. **Meredith McMorrow:** funding acquisition, writing–review and editing. **Wesley H. Self:** writing–original draft, writing–review and editing, supervision, funding acquisition, project administration, data curation, methodology.

## Disclosure

The findings and conclusions in this report are those of the authors and do not necessarily represent the official position of the Centers for Disease Control and Prevention (CDC).

## Conflicts of Interest

All authors have completed and submitted the International Committee of Medical Journal Editors form for disclosure of potential conflicts of interest. Samuel Brown reports that ReddyPort pays royalties for a patent, outside the submitted work. Steven Chang reports consulting fees from PureTech Health and Kiniksa Pharmaceuticals, outside the submitted work. Abhijit Duggal reports participating on an advisory board for ALung Technologies, outside the submitted work. Manjusha Gaglani reports grants from CDC, CDC‐Abt Associates, CDC‐Westat, and served as co‐chair of the Infectious Diseases and Immunization Committee for the Texas Pediatric Society (TPS) and received an honorarium serving as a TPS Project Firstline webinar speaker panelist for “Respiratory Virus Review: Clinical Considerations and IPC Guidance,” outside the submitted work. Michelle N. Gong reports a grant from NHLBI and CDC, fees for serving on Scientific Advisory Panel for Philips Healthcare, travel to ATS conference as board member, outside the submitted work. Carlos Grijalva reports grants from NIH, CDC, AHRQ, FDA, and Syneos Health and receipt of compensation for participation in an advisory board for Merck, outside the submitted work. Natasha Halasa reports receiving grants from Sanofi, Merck, and Quidel, outside the submitted work. Adam Lauring reports receiving grants from CDC, FluLab, NIH/National Institute of Allergy and Infectious Diseases, Burroughs Wellcome Fund, and MDHHS and consulting fees from Roche related to baloxavir, outside the submitted work. Christopher Mallow reports Medical Legal Consulting, outside the submitted work. Ithan D. Peltan reports grants from NIH and Janssen Pharmaceuticals and institutional support Regeneron, outside the submitted work. Mayur Ramesh reports participating in a non‐branded Speaker Program supported by AstraZeneca and MD Briefcase and participating on an advisory board for Moderna, Pfizer, and Ferring, outside the submitted work. No other potential conflicts of interest were disclosed.

### Peer Review

The peer review history for this article is available at https://www.webofscience.com/api/gateway/wos/peer‐review/10.1111/irv.70027.

## Supporting information


**Appendix S1** Investigators and Collaborators.
**Appendix S2**. Supplementary Methods.
**Appendix S3.** Supplementary Figures and Tables.
**Figure S1.** Participant flow diagram.
**Figure S2.** Number of COVID‐19 case patients and sequenced SARS‐CoV‐2 lineages by admission week during 8 September 2022–31 August 2023.
**Figure S3.** Relative bivalent vaccine effectiveness against COVID‐19‐associated hospitalization among adults in the United States during 8 September 2022–31 August 2023.
**Figure S4.** Relative bivalent vaccine effectiveness against COVID‐19‐associated severe in‐hospital outcomes among adults in the United States during 8 September 2022–31 August 2023.
**Table S1.** Sensitivity analyses for covariate selection.

## Data Availability

No additional data are available.
